# Hair transplantation surgery

**Published:** 2008-10

**Authors:** Manoj Khanna

**Affiliations:** Consultant Cosmetic Surgeon, Cosmetic Surgery Clinic, 12, Loudon Street, Suite 3C, Kolkata-700 017, India

**Keywords:** Hair transplantation, Follicular unit, graft, Kolkata slit

## Abstract

Techniques in hair transplantation have evolved recently which make results look more natural. Hair restoration is one of the most exciting and innovative surgical fields in aesthetic surgery today. A precise appreciation of anatomy has allowed the use of follicular unit grafts. With better methods of harvesting and implantation, hair transplantation results represent a blend of art and science.

## INTRODUCTION

Hair transplantation is one of the most rapidly evolving procedures in aesthetic surgery, accompanied by regular improvement in techniques. The recent advances in technology and the concept of using *follicular unit grafts* have made this procedure reach a new height. The ability to provide very natural-looking results has encouraged larger number of balding men and women to opt for this surgical solution.

## PATHOPHYSIOLOGY

The clinical onset of baldness in both men and women is generally around the age of 30 to 40 years. A strong family history is one of the best indicators of male pattern baldness or androgenic alopecia, which is the most common cause of hair loss. An autosomal dominant genetic linkage is believed to cause this hair loss. Male pattern baldness may begin in the teen years, and becomes more common with increasing age. It is known that the male hormone, testosterone, gets converted to another male hormone, 5-dihydroxytestosterone (5-DHT), in the hair follicles. Under the influence of 5-DHT, hair follicles in the front and the top of the scalp begin to become more fine over the years in genetically susceptible men. Hair growth also gets restricted and eventually the hair disappears completely.

**Figure 1:**
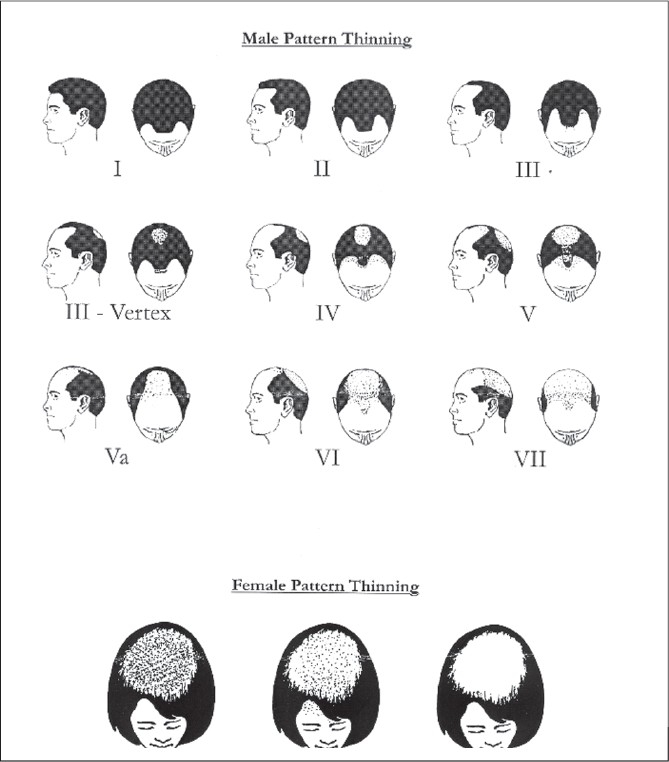
Hair loss classification

Like most tissues, hair undergoes a continuous turnover throughout life. Hair follicles are replaced periodically, and at any given time, they are in one of three stages of their growth cycle. The actively growing stage (*anagen phase*) is followed by a brief period of morphological change or the involution stage (*catagen phase*). This is then followed by a resting stage (*telogen phase*). In normal human beings, the total number of scalp hair is usually 100,000. Hair grows at the rate of 1–2 cm every month and the duration of the anagen phase is 2–4 years while that of the telogen phase is 100 days. Approximately 40–100 hairs are shed daily; this rate increases in late summer and early autumn, and decreases in late winter or early spring, due to the effects of temperature. Norwood has classified baldness into seven stages [Figure 1]. In women, the frontal hairline is usually spared and baldness in females has been classified separately by Ludwig.

Hair transplantation is based on the *‘theory of donor dominance in androgenic alopecia.’* If a graft is taken from an area destined to be permanently hair-bearing and transplanted to an area suffering from male patterns baldness, it will, after an initial period of effluvium, grow hair in its new site as long as it would have at its original site. This is the scientific basis of hair transplantation surgery.

## TERMINOLOGY

Terminal hair is androgen-dependent, male-type hair on the face (mustache, beard, and sideburns) and on the body (chest, areola, linea alba, inner thighs). It increases in hirsutism. Vellus hair is nonpigmented, fine “peach fuzz” hair covering the body in both children and adults. It increases in hypertrichosis.

The *follicular unit graft* (FUG) as described by Headington[1] includes 1–4 terminal hair follicles, one (or rarely two) vellus follicles, associated sebaceous lobules, insertion of erector pili muscle, perifollicular neurovascular network etc. This definition suggests that the unit is a physiological entity rather than an anatomical one. For all practical purposes, it is best to describe a *follicular unit* as an aggregation of hair shafts emerging from the scalp, in which the distance between the hairs is less than the distance to the nearest aggregation of hairs. This pattern has to be kept in mind while harvesting, dissecting, and transplanting hair to achieve maximal efficiency, and to give a natural appearance to the patient.

## HAIR TRANSPLANTATION TECHNIQUE

### Planning

Although age is no bar for hair transplantation, the pros and cons of a transplant need to be carefully evaluated in the younger patients. Patients between 20 and 30 years of age should have a stabilized rate of hair loss before they are considered for hair transplantation. A detailed family history is useful in assessing hair loss and planning a new hairline.

The colour, quality, and density of the donor hair, as well as the contrast between the hair and the skin colours, are important factors that affect the result. The lesser the contrast between the donor hair and the skin, the better is the result.[2] It is also noted that frizzy, curly, or wavy hair are advantageous characteristics in transplanted hair.

Single hair grafts are used to create a natural hairline. The planning of the hairline is one of the most important steps in hair transplantation. The hairline is the most visible landmark and the quality of work of a surgeon is often judged by the quality of the hairline. As suggested by Michaelangelo, to locate the ideal hairline in a bald patient, it is necessary to divide the face into three equal segments.[3] In the midline, the hairline starts at least 8 cm from the glabella. A curve sweeps around to the lateral side of the forehead from the center. At this point, the sides of the hairline should be oriented parallel to the curve when the subject is looking straight ahead. The lateral hairlines are usually 9.5–11.5 cm above the lateral canthus of the eyes. The temporal angles should form relatively sharp right angles or acute angles in most men, but these angles should be more rounded in women. The hairline shape also varies according to the variation of the shape of the face—round, oval or triangular. The patient's desires and constraints are also other factors that can affect the shape of the hairline.

Usually 250–300 single hair (micro)grafts will be necessary to create a new hairline in any individual. The micrografts in the hairline should be placed in an irregular saw-toothed pattern of macro- and microirregularity[4] to give a natural appearance. Behind the hairline, two-hair FUGs are used to provide new hair. Three or four hair FUGs are used just further behind. The less ideal the hair and skin characteristics, the more important it is to use smaller grafts. To give good density in alopecic recipient areas, some surgeons use punch grafts that are 1, 1.25, and 1.5 mm in diameter, behind the hairline. The punch grafts have the advantage of removing a circular area of bald tissue where the grafts will be placed. These punch grafts should not be used in areas where hairs are already present as they would punch out existing hair and the surgery will be counterproductive. Punch grafts can however, be useful in areas of total baldness.

### Preoperative preparation

The patient is asked to shampoo his head with Betadine surgical scrub on the day before, and on the morning of the surgery.

### Preparation of the donor area

Local anaesthesia is used for the entire procedure. A solution is made from 30 mL of 2% lignocaine with 100 mL of normal saline, to which 1 mL of adrenaline (1:1000) is added. The hair in the donor area (occipital region) is trimmed to a length of 2–4 mm and the local anaesthetic solution is injected just below the donor area. The donor area is then tumesced by injecting normal saline into the entire zone. After 10–20 minutes for complete haemostatic effect to minimize bleeding, the donor area should be turgid at the completion of infiltration, because this provides excellent anaesthesia and results in minimum bleeding.

### Harvesting

The donor strip can be harvested with a single-bladed knife or a multiple-bladed knife containing three to seven blades. The multibladed knife harvests numerous (two to six) parallel strips of varying width (depending on the spacer used), which may be 1.5, 2, or 2.5 mm. These blind incisions with a multibladed knife increase the chance of follicular damage; therefore, it is better to use a single or a double-bladed knife. It is very important that while harvesting the donor area, the blades remain parallel to the direction of the hair so that the hair roots are not damaged. The hair in the lower part of the occipital area and the temporal area are finer, and these should be used to create a new hairline. After the strip has been harvested, the gap can be closed either with staples or sutures. Some surgeons, including the author, prefer deep sutures in the galea or the subcutaneous tissue to reduce the width of the scar. The skin can be opposed by a running suture of 3-0 or 4-0 monofilament nylon, or any absorbable suture. Care is taken to take the bites close to the skin margin to avoid more damage to the tissues. Also, it is important to take the bites only up to the dermis so that the deeper hair roots are not damaged, and thus can be utilised in subsequent surgery.

Follicular Unit Extraction[5] is a technique that involves the removal of the intact follicular unit directly from the donor area using a 1 mm punch. The yield by this harvesting technique can decrease due to transection and avulsion injury to the follicular unit. Also, although marketed as a technique that leaves no scar in the donor area, it leaves multiple ‘dot-scars’ in the donor area, which are larger than those left by the strip method.

Harvesting donor hairs which are white or light coloured is more difficult. Extra care has to be taken to preserve the hair follicles. To enhance visibility, these patients are instructed to dye the hair a few days before the procedure. Methylene blue can be injected in a very low concentration just before surgery into the donor area to help in identifying the grey hairs during dissection. Extra care is also required in patients undergoing a second procedure because scars from previous surgery, distort the direction of the hair in the donor area.

### Graft preparation

The harvested donor strips are immediately immersed in chilled normal saline. This is achieved by keeping the tray containing the grafts, immersed in saline on ice. Proper hydration of the donor grafts with cold saline is very important throughout the surgery as it influences the survival rate of the grafts. If a single large strip has been harvested, it can be divided into smaller pieces or slivers[6] before the cutting of individual grafts. The subcutaneous fatty tissue below the hair roots or bulbs is stripped leaving up to 2 mm of fat below the hair bulb. FUGs are made having one to four hairs. Grafts are immersed in saline in a Petridish, or kept on a moist stockinet in kidney trays, in bunches of 25. Good illumination is essential during the cutting of the grafts. The grafts may be cut on wooden tongue depressors or on a clear vinyl dissecting surface with a backlighting system. It is important that no piece of wood sticks to the grafts after they have been cut, because these foreign bodies can later form troublesome epidermal cysts. Loupe magnification of 2X or 3X power is useful in creating FUGs. Graft preparation with a dissecting stereo microscope makes the dissection a little slower, but it is much more accurate. Some surgeons prefer slicing the epidermis in the grafts at an angle of 45° to avoid scab visibility in the postoperative period, but this takes more time and is not preferred by the author.

### Preparation of the recipient area

Anaesthesia for the recipient area includes a supratrochlear and supraorbital nerve block, followed by a ring block in the frontal area beyond the zone of hair transplantation. The recipient area itself should be tumesced well with normal saline. It is the author's preference to avoid using adrenaline in the recipient area because it increases telogen effluvium in the immediate postoperative period, and it also may diminish the uptake of the grafts. Adrenaline must definitely be avoided in the recipient area in women[7] because severe effluvium has been reported after its use. To minimize bleeding and pain, the recipient area should be turgid before slits or holes are made.

While making slits or holes in the recipient area, it is very important to follow the direction of the existing hair in that region. The hairline should have a ragged, saw-toothed natural look. Holes are made with a No. 18 / 20/ 23 gauge needle in a pattern of organized disorganization. About 250–300 micrografts are necessary to create a normal hairline. Behind the hairline, slits can be made by Nokor® needles, a Minde® knife (A – Zee Surgical, USA), a No. 11 scalpel blade or by needles. The author has devised a new instrument which is being patented as “Kolkata slit”. The Nokor needles and Minde knife are disposable instruments and not easily procurable in India. The scalpel blades make holes that are too large and often deep, because of which the inserted grafts float and lose direction. Scalpel blades can also cause significant damage to the existing hair in the recipient area. In females, a large number of hair strands get cut by the scalpel blade during the procedure. The ‘Kolkata slit’ is an instrument which can be re-used and comes in different sizes. It creates a gap just about the size of the graft to be inserted, and ensures that the graft maintains the direction of orientation. The slit may be used in attempts to increase density in areas where there are existing hairs.

In patients undergoing secondary or tertiary procedures, an increased amount of bleeding has been noticed in the recipient area. Increased bleeding is also seen in patients who have been using minoxidil lotion in the preoperative period. Good tumescence and a waiting period of 10–15 minutes before making gaps can reduce this disturbing ooze. It is also noticed that the gaps in recipient areas are tougher to make in secondary procedures, because of fibrosis from earlier procedures.

### Graft insertion

The grafts are placed into the recipient slits / holes using fine-angled forceps. It is important to employ an atraumatic technique for graft placement. To avoid damage, the FUGs are grasped by the 2 mm of subcutaneous tissue left below the hair bulbs to position them into the recipient sites and not by the follicle end. A steady pressure is applied to ensure that the grafts are flush with the surrounding skin. Burying the grafts beneath the level of the skin must be avoided because it can give a pitted appearance and also lead to the formation of epidermal cysts. A cobblestone appearance is seen if the grafts are too elevated from the surface. Two, or even three, persons can insert grafts at the same time to make the procedure faster and efficient. Grafting sessions can last up to five or six hours, in which 2000–3000 FUGs may be transplanted.

### Postoperative care

The patient is discharged the same day, usually without any bandage. Some surgeons still prefer to bandage but it must be done very carefully to avoid shearing. The bandage must also be removed very meticulously because grafts can stick to the undersurface and get removed inadvertently.

Some swelling is obvious after a hair transplantation surgery and the patient should be informed of this prior to the procedure. Oral steroids for 3–5 days can minimize the oedema. Some surgeons use Injection Triamcinolone 40 mg in the tumescent solution and claim that this reduces the swelling. A head-band worn immediately after the operation is useful in preventing the swelling from coming down on to the face and creating a puffy appearance. The patient is instructed to wash his hair with a mild shampoo on the 2^nd^ or 3^rd^ postoperative day. While combing the hair in the transplanted area for three weeks, the tooth of the comb should not strike against the transplanted grafts. Wearing clothes like T-shirts or pullovers which have to be taken off over the head should also be avoided for three weeks. Hair oils or other stronger shampoos as well as helmets are also to be avoided for the same period. In men, 5% minoxidil lotion is applied in the areas of the hair transplant once the shampooing has begun while 2% minoxidil lotion is used in females. This is continued for a period of two to six months. This has been shown to promote earlier growth of the transplanted hair.

### Sequel

The epidermis and dermis along with the shaft of the transplanted hair outside the skin fall off as scabs in the two to three weeks after the surgery, but the follicles remain and go into a resting phase. New hairs start growing about three months after the procedure. It has often been noticed that with the use of 5% minoxidil, the hairs do not fall and start growing immediately in the postoperative period. It usually takes six to nine months to appreciate the result of a hair transplant. If a second procedure has been planned, it must be at least three to six months after the first sitting. Some patients may complain of hypoaesthesia of the scalp in the donor area. It is usually temporary, but may persist for as long as 18 months in some cases.

The density of transplanted hair is thinner especially in areas that are totally bald. The patient should be informed of this preoperatively and a second sitting can be undertaken to increase hair density.

### Complications

Complications of hair transplantation are few and rare. True infections in the recipient areas occur infrequently. In the donor areas, infection may be seen around the sutures but it usually resolves easily after suture removal. Epidermal cysts may be seen occasionally and need drainage. It is important not to harvest too big a donor area because tension on the suture line can lead to dehiscence and a wide scar.

### Hair transplants in special sites

Eyebrow transplantation can be done to improve or recreate eyebrows. It is an aesthetic essentiality to follow the direction of the eyebrow hairs while creating a new line. Around 150 micrografts are usually required for an eyebrow of one side. The donor site for eyebrow transplantation should be of finer hair preferably from the nape of the neck or the temporal region.[8] Recipient holes are made with a No. 20 or 21-gauge needle or a 0.7 mm microblade. Cyanoacrylate glue may be used over the grafted areas to keep the grafts in place during the immediate postoperative period.

Grafting eyelashes is a more challenging procedure. Fortunately, only a few lashes are necessary to produce a good result. Six one-hair micrografts per lid may satisfy most patients. Cyanoacrylate glue is again very useful in keeping the grafts in place.

The rate of hair growth of the scalp hair is much faster than those of the eyebrows and elsewhere. Patients must be informed preoperatively that this transplanted hair will need trimming from time to time.

Moustache reconstruction by hair transplantation is especially useful in patients who have had a cleft lip or a scar following trauma. The hair in the moustache area is much more wiry and coarser than hair in the scalp. Harvesting hair from the beard area just inferior to the jaw line may provide better donor hair for moustache reconstruction.[9]

Patients who have undergone hair transplantation using older techniques have larger plugs. This gives the hairline a pluggy, corn-row appearance that needs correction. The current approach uses plug reduction and recycling, and is applied aggressively to the front two rows.[10]

## CONCLUSION

Recent advances in technology have made hair replacement surgery a viable option for many people but we must utilize this technique prudently. It is very important to form a team because one individual cannot perform the entire procedure single-handedly. Fine tuning and accuracy in all steps of the surgery are essential to get good results. No compromise should be made with proper lighting in the operating room and with the quality of the instruments. A comfortable ambience in the operating room and use of audio-visual entertainment break the monotony, both for the patient and the surgical team.

It is important to remember that a patient is worse off after a poorly performed hair replacement surgery. If done judiciously, transplantation is a very rewarding procedure, both for the surgeon and the patient.

**Figure 2:**
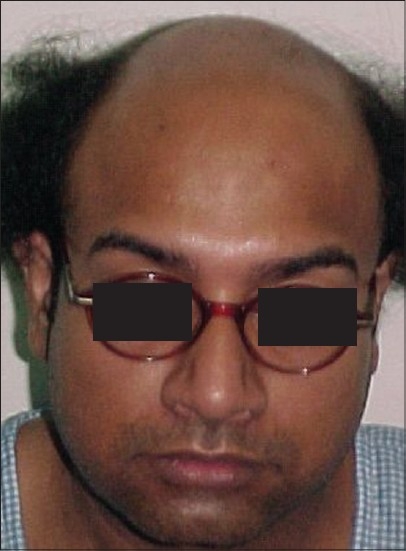
AG- Pre-Hair Transplant (HT)

**Figure 3:**
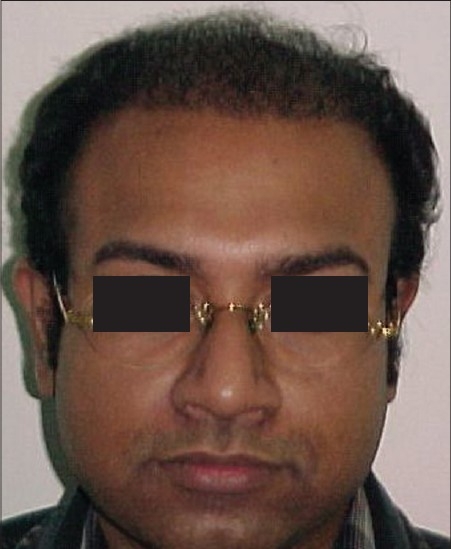
AG-Post-HT of 2000 FUGs

**Figure 4:**
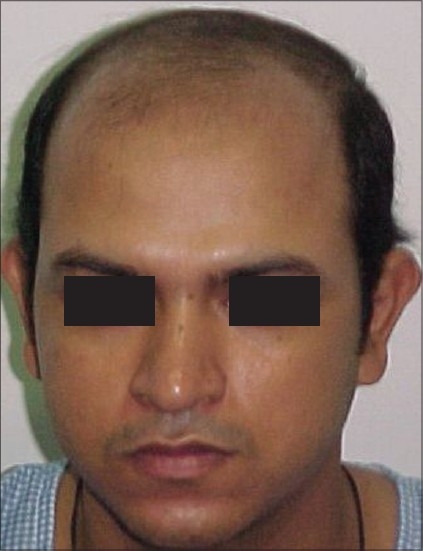
BD- Pre-Hair Transplant (HT)

**Figure 5:**
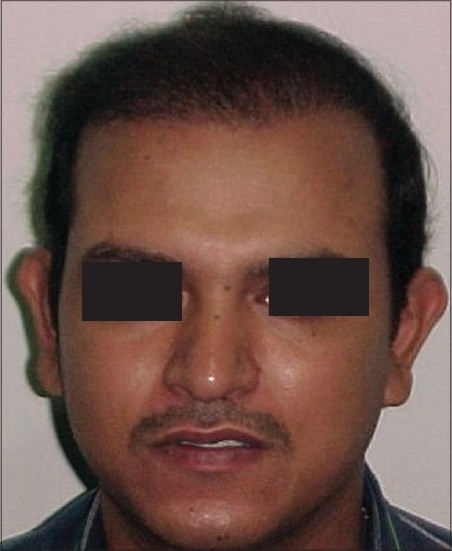
BD-Post-HT of 1800 FUGs

**Figure 6:**
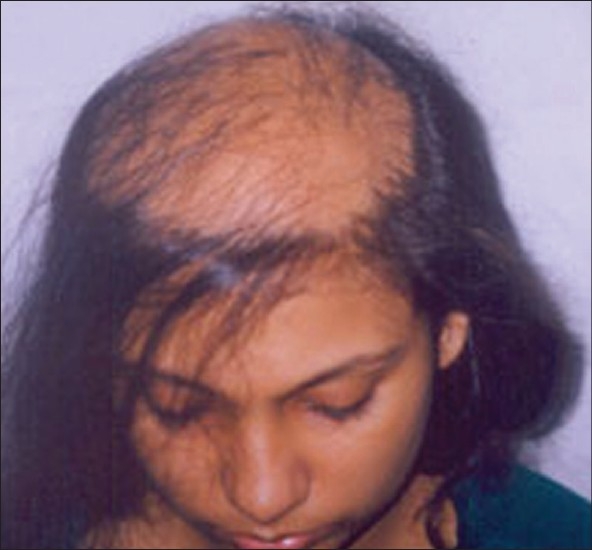
JM- Pre-Hair Transplant (HT)

**Figure 7:**
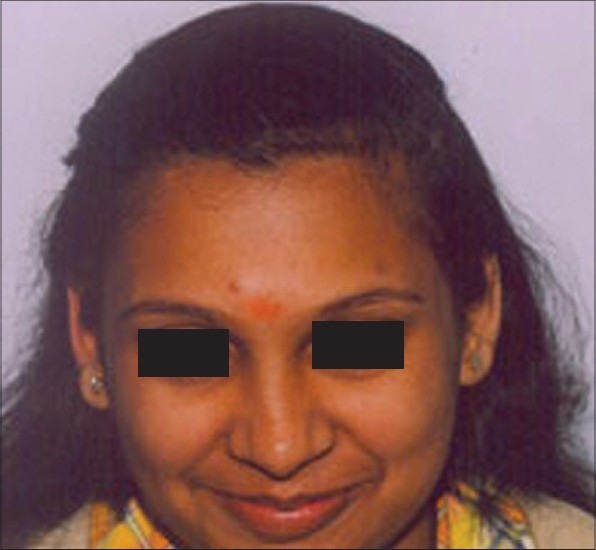
JM-Post-HT of 2100 FUGs

**Figure 8:**
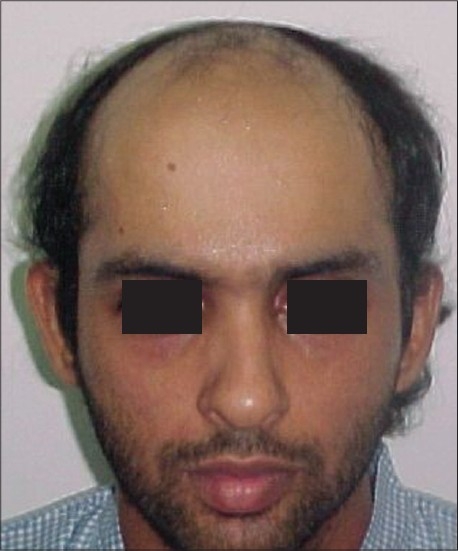
VB- Pre-Hair Transplant (HT)

**Figure 9:**
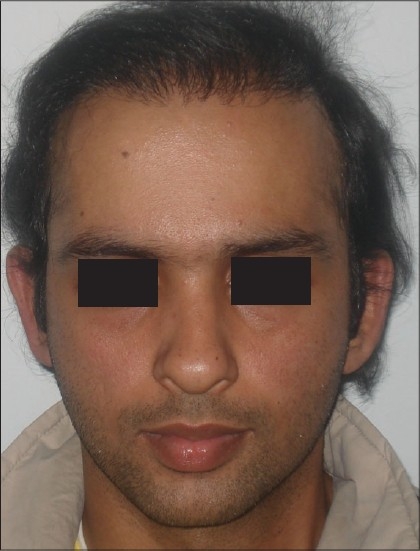
VB-Post-HT of 1900 FUGs

**Figure 10:**
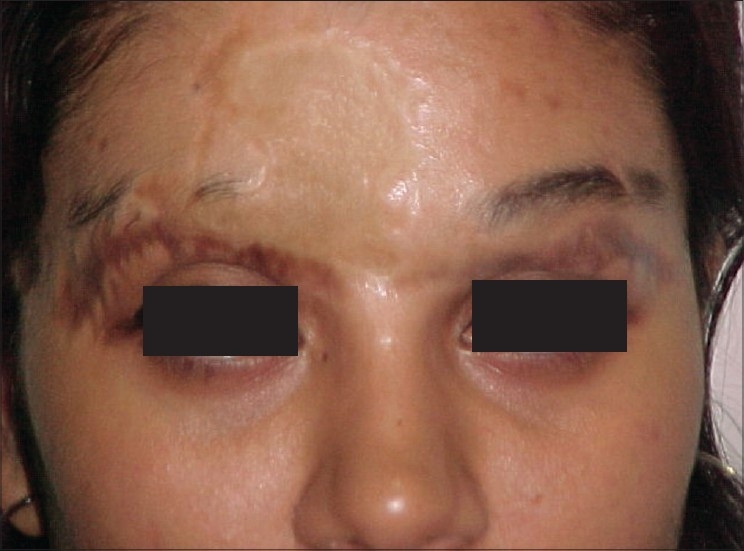
SK- Pre-Hair Transplant of Eyebrows

**Figure 11:**
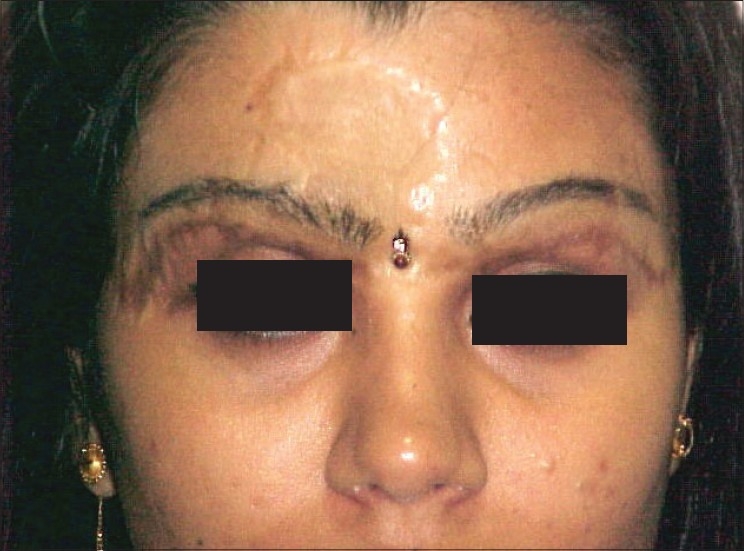
SK- Post-Hair Transplant of Eyebrows
